# Metasurface spatial filters for multiple harmonic signals

**DOI:** 10.1515/nanoph-2022-0752

**Published:** 2023-03-31

**Authors:** Daeik Kim, Mai Anh Nguyen, Gangil Byun, Jongwon Lee

**Affiliations:** Department of Electrical Engineering, Ulsan National Institute of Science and Technology (UNIST), Ulsan 44919, Republic of Korea

**Keywords:** beam steering, harmonic frequencies, metasurfaces, spatial filters

## Abstract

Nonlinear frequency mixings have shown an alternative way to create new electromagnetic sources in frequency ranges that are difficult to access with conventional techniques. To simultaneously use the fundamental frequency pump beam and multiple harmonic signals generated in the same channel, a device capable of separating each frequency component is required. Here, we propose and experimentally demonstrate metasurface-based spatial filters for the pump frequency and multiple harmonic frequencies. The metasurface was designed using eight different split ring resonator-based phase elements with 45° phase spacing, which allows wavefront shaping. The metasurface designed to have a one-dimensional gradient phase array produces cross-polarized reflection waves with different beam steering angles at the third- and fifth-harmonic frequencies (15 and 25 GHz) and operates as a metallic mirror at the fundamental frequency of 5 GHz. Our work suggests a new method to enable simultaneous use of broadband multi-frequency sources based on nonlinear frequency mixing.

## 1 Introduction

Frequency mixings based on nonlinear optical phenomena have been suggesting an alternative to developing new frequency light sources that are technically difficult to generate. In particular, studies using nonlinear optical responses have been conducted to develop light sources in the frequency range of 0.1–10 THz, called the THz gap. Recently, the possibility of using giant odd-order nonlinear optical susceptibilities due to the nonlinear conductance in monolayer graphene has been reported [1–4], and studies on odd-order harmonic generations using graphene monolayer have been reported [5, 6]. Due to the centrosymmetric structure of graphene, even-order harmonics cancel out and only odd-order harmonics *f*, 3*f*, 5*f*, …, can be generated [7]. In such system where multiple harmonic generations occur, a device capable of separating each harmonic frequency component is required to simultaneously use several frequency components including a fundamental frequency pump beam. One way to meet this requirement is to use a spatial filter for multiple harmonic frequencies that can spatially separate each frequency component.

Metasurfaces, consisting of an two-dimensional arrangement of subwavelength metallic or dielectric structures called meta-atoms, exhibit an ability to engineer the amplitude, phase, and polarization of scattered light from meta-atoms at a deeply subwavelength scale [8, 9]. Recently, the local control of the scattered light from metasurfaces have shown a new way to engineer the wavefront shaping and the beam-steering, leading to a new concept of “flat optics” that can replace conventional bulk optics. Based on the unique properties of metasurfaces, interesting applications of flat optics have been demonstrated such as flat lens [10], waveplate [11–13], and hologram [14, 15]. With periodical or non-periodical arrangements of meta-atoms, broadband metasurfaces for beam steering and beam forming have been studied [16–18]. The local phase and amplitude response of individual meta-atom can be adjusted by the designed structural parameters under the linearly polarized light. However, it is hard to cover fundamental frequency (FF) to high harmonic frequencies (HFs) due to the limitation of operation bandwidth by the intrinsic dispersion of resonance from meta-atoms [19–21].

In this work, we propose and experimentally demonstrate metasurfaces for spatially separating fundamental frequency (*f*) and two odd-high harmonic signals (3*f* and 5*f*). For the proof-of-concept demonstration, we used fundamental frequency of 5 GHz, third- and fifth-harmonic frequency of 15 and 25 GHz. The metasurface consist of split ring resonators with different gap sizes in reflection mode to control the phase and amplitude of electromagnetic (EM) wave at a broadband frequency range. In this work, we propose a new approach of using polarization conversion of meta-atom to overcome the bandwidth limitation. The polarization state of reflected wave from split ring-shaped particle can be controlled by changing structural parameters under linearly polarized wave. To cover the FF to high HFs, different polarization states were used for FF and high HFs. In this configuration, polarization state of the reflected wave at FF was designed to be the same as state of incident wave, and polarization states at high HFs were designed to be opposite state of the incident wave polarization. Each polarization states were affected on different resonances; therefore, the operating bandwidth can be tuned by the two resonances. This notable feature guarantees a wide bandwidth for engineering of local phase and amplitude that can cover FF to high HFs.

## 2 Multi-frequency beam steering metasurface design

**Figure 1:**
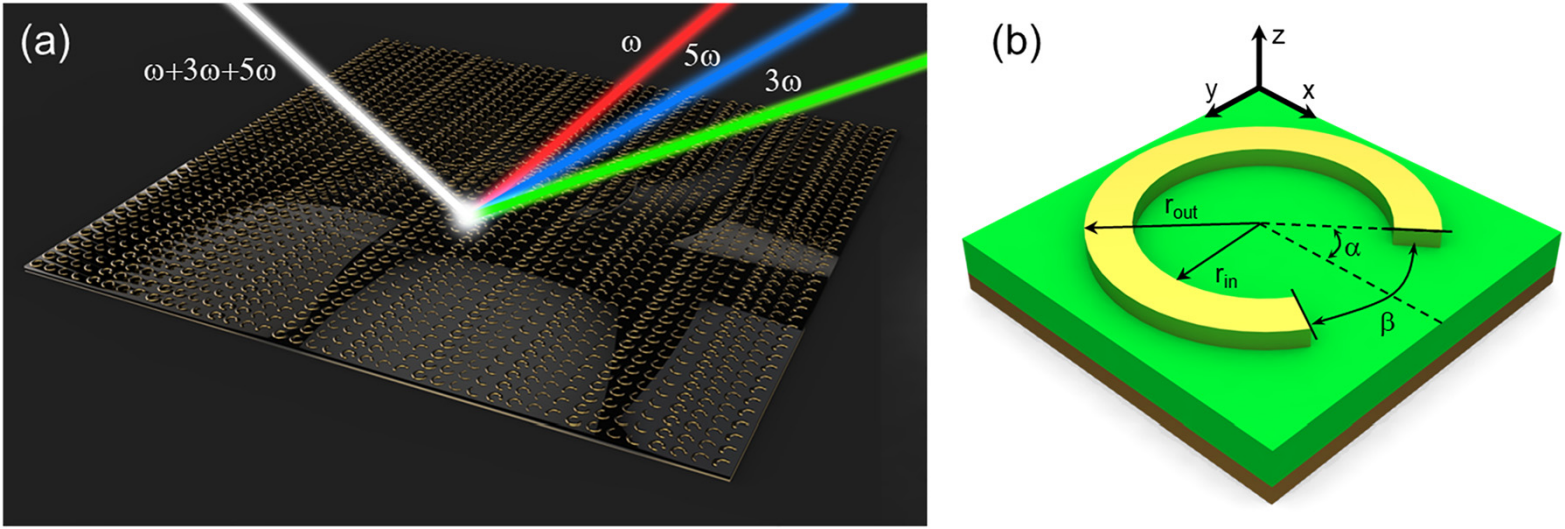
Multi-frequency beam steering metasurface. (a) Schematic illustration of the multi-frequency beam steering metasurface. The white beam indicates an incident beam with three frequency components of *ω* (FF), 3*ω* (THG), and 5*ω* (FHG). The red, green, and blue beam indicates reflected beams from the metasurface at FF, THG, and FHG, respectively. (b) Unit meta-atom structure with SRR. Local phase and amplitude of scattered wave from the meta-atom are controlled independently by varying the angles of *α*, *β* and the inner and outer radius of SRR, *r*_in_, and *r*_out_, respectively.

Figure 1(a) shows a schematic of our multi-frequency beam steering metasurface that consists of split ring resonator (SRR) arrays designed at 5 GHz, 15 GHz, and 25 GHz, effectively working as a multi-frequency spatial filter. The SRR is a well-studied as a constituent meta-atom to control the local phase and it is polarization sensitive under the linearly polarized beam [17, 22]. Through optimization of SRR structure, we obtained SRR designs covering a broadband frequency range for high harmonic generation signals with reflection coefficients of nearly 1 using the polarization sensitive property. A unit cell design of the split ring resonator is shown in Figure 1(b). The split ring resonator arrays were patterned on a dielectric substate layer on a metal back plane. The dielectric substrate material was Taconic TLT-6 with a dielectric constant of 2.65, a loss tangent of 0.0006, and thickness of 2 mm. The phase and amplitude properties of reflected beam from the metasurface were tuned by adjusting the angle of α and β and the inner and outer radius of the SRR, *r*_in_ and *r*_out_ as shown in Figure 1(b). The design parameters of the SRR are provided in Table 1.

**Table 1:** Design parameters of the eight meta-atom unit structures.

No. of meta-atom	1	2	3	4	5	6	7	8
*r*_in_ (mm)	1.5	1.5	1.5	1.5	1.5	1.5	1.5	1.5
*r*_out_ (mm)	2	2	2	2	2	2	2	2
*α* (°)	45	45	45	45	−45	−45	−45	−45
*β* (°)	50	94	137	170	50	94	137	170

**Figure 2:**
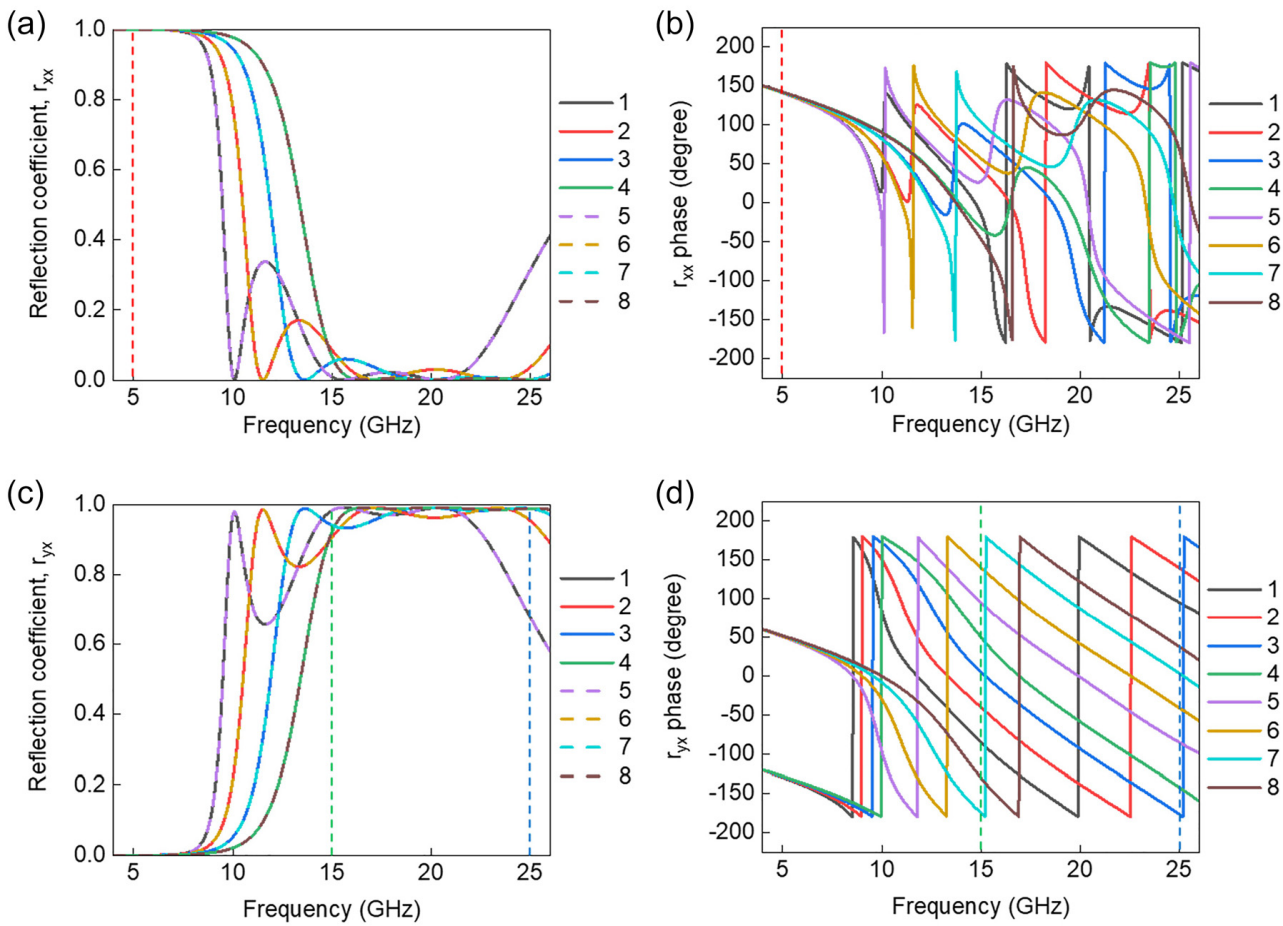
Simulated reflection coefficients of the eight meta-atoms. (a) and (b) Simulated (a) magnitude and (b) phase spectra of reflection coefficient for *x*-polarized reflection beam under the *x*-polarized incident beam, indicated as *r*_*xx*_. (c) and (d) Simulated (c) magnitude and (d) phase spectra of reflection coefficient for *y*-polarized reflection beam under the *x*-polarized incident beam, indicated as *r*_*yx*_.

Figure 2 shows the simulated local phase and reflection coefficient of the designed meta-atoms using by Commercial Software package (CST Microwave Studio). For wavefront engineering, eight different meta-atoms were designed and arranged to form a super cell. Figure 2(a) and (b) shows simulated magnitude and phase of reflection coefficients of the eight meta-atoms under the *x*-polarized input and the same polarized reflection output, indicated as *r*_*xx*_. In case of the *r*_*xx*_ at 5 GHz, the reflection coefficients are nearly unity and reflection phase values are the same for the eight meta-atoms. Therefore, the super cell acts as a simple metallic mirror, leading to specular reflection at 5 GHz for the *r*_*xx*_ component. Figure 2(c) and (d) shows simulated magnitude and phase of reflection coefficients of the eight meta-atoms under the *x*-polarized input and *y*-polarized reflection output, indicated as *r*_*yx*_. In case of the *r*_*yx*_, reflection coefficients are over 0.9 and 0.65 at 15 GHz and 25 GHz, respectively, for the eight meta-atoms and local phase values form eight different values with nearly 45 degrees of spacing at the two harmonic frequencies. Therefore, any arbitrary wavefront engineering is possible by using the eight phase element meta-atoms at the two harmonic frequencies. As shown in Table 1, four of two pairs of meta-atoms (1&5, 2&6, 3&7, and 4&8) have the same shape with a different in-plane orientation angle of 2*α* = 90°, where the cross-polarized reflection coefficient has the same magnitude as *r*_*yx*_, but there is a phase difference by *π*. To build spatial harmonic frequency filters, we designed a super cell structure with different beam steering angles by producing local phase gradients along the long axis of the super cell at 15 GHz and 25 GHz. According to the generalized Snell’s law, beam steering angle of a gradient phase metasurface can be expressed as [20]:

(1) $$\theta_{\text{steering}}=-\arcsin\left[\left(\Delta \phi /360{}^{\circ}\right)\lambda /L\right]$$

where *ϕ* is the phase difference between adjacent meta-atoms, *λ* is wavelength of incident EM wave and *L* is the lateral length of the unit cell. The calculated beam steering angles for the two harmonic frequencies using Equation (1) were −30° for 15 GHz and −17° for 25 GHz. The large beam steering angle difference for the two harmonic frequencies from the single super cell structure is mainly obtained by the large wavelength difference when properties of high reflection coefficients and uniform phase spacing at the two frequencies are guaranteed.

## 3 Numerical and experimental results

**Figure 3:**
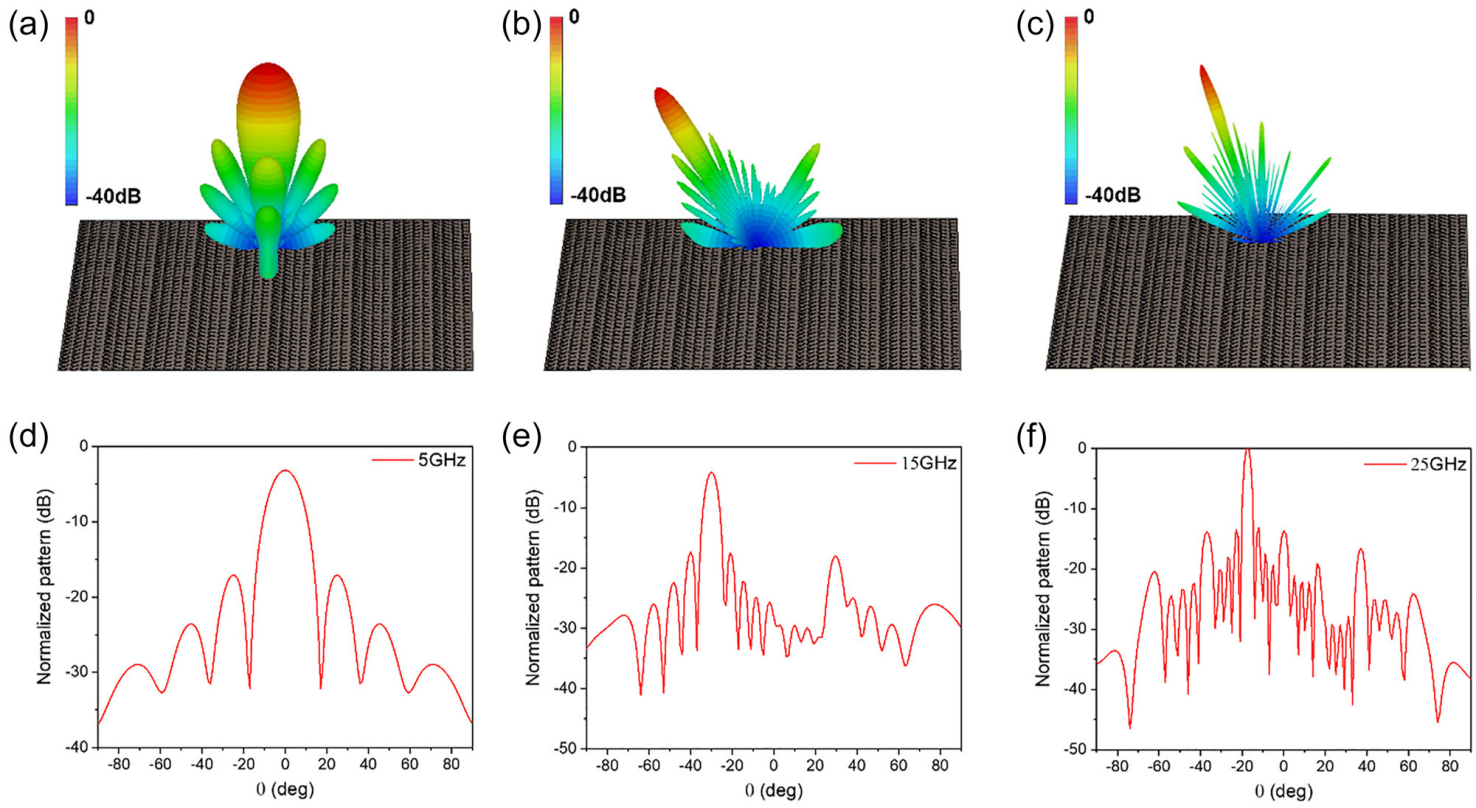
Simulation results of far-field scattering patterns from the metasurface. (a)–(c) Three dimensional scattered intensity patterns at (a) 5 GHz, (b) 15 GHz, and (c) 25 GHz under normal incidence wave. (d)–(f) Two-dimensional cross-section of the scattered intensity patterns at (d) 5 GHz, (e) 15 GHz, and (f) 25 GHz under normal incidence wave.

Far-field radiation pattern simulations were conducted to confirm the steering angle of reflected beam from the designed metasurface under a normal incidence of a plain wave. Figure 3 show the 3D and 2D cross-section of simulated far-field radiation patterns at 5 GHz, 15 GHz and 25 GHz. The intensity radiation patterns at each frequency were normalized to the respective maximum intensity value for clear identification. At 5 GHz, the perpendicularly incident beam on the metasurface is reflected vertically as shown in Figure 3(a) and (d) and acts as metallic mirror. For normal incident waves at 15 GHz and 25 GHz, the beam angles reflected from the metasurface are steered to −30° and −17°, respectively. The numerical simulated and calculated results are matched very well. Therefore, when beams of three frequency components of 5, 15 (THG), 25 GHz (FHG) are incident vertically on the designed metasurface, they are steered to different reflection angles of vertical, −30° and −17°, respectively, and through the characteristics, the metasurface operates as a spatial frequency filter.

**Figure 4:**
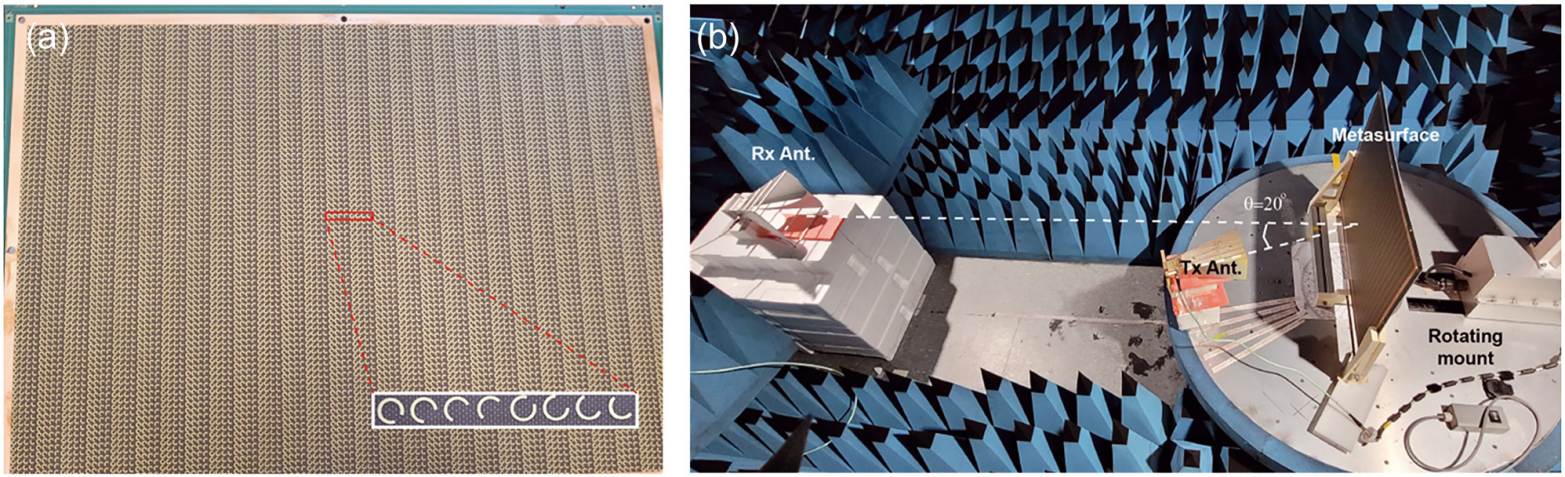
Processed metasurface and measurement setup. (a) Photograph of the fabricated sample and (inset) zoom-in view of the super unit cell image. (b) Experimental beam steering measurement setup consisting of the transceiver horn antenna (Tx Ant.), the metasurface, the receiver horn antenna (Rx Ant.) and the rotating mount.

For experimentally demonstration of the proposed approach for multi-frequency beam steering, the designed metasurface was fabricated by using standard printed circuit board (PCB) process as shown in Figure 4(a). The thickness of substate is 2 mm and the size of metasurface is 600 mm × 400 mm. Figure 4(b) shows far-field measurement system. Scattering patterns reflected from the metasurface was measured in anechoic chamber to avoid background noise signals. In measurements, two different polarization-sensitive horn antennas were used to produce the two linearly polarized incident beams. The transceiver horn antenna (Tx antenna, *x*-polarized state) was tilted at +20° to avoid interference due to reflected signals, and the distance between the Tx antenna and the metasurface was set to 330 mm. The distance between receiver horn antenna and the metasurface was set to 2 m for collecting far-field radiation patterns. By rotating the mount of the Tx antenna and the metasurface with a 0.5° step, far field profiles of reflected signal from metasurface at the three frequencies were measured using the two ports antenna system.

**Figure 5:**
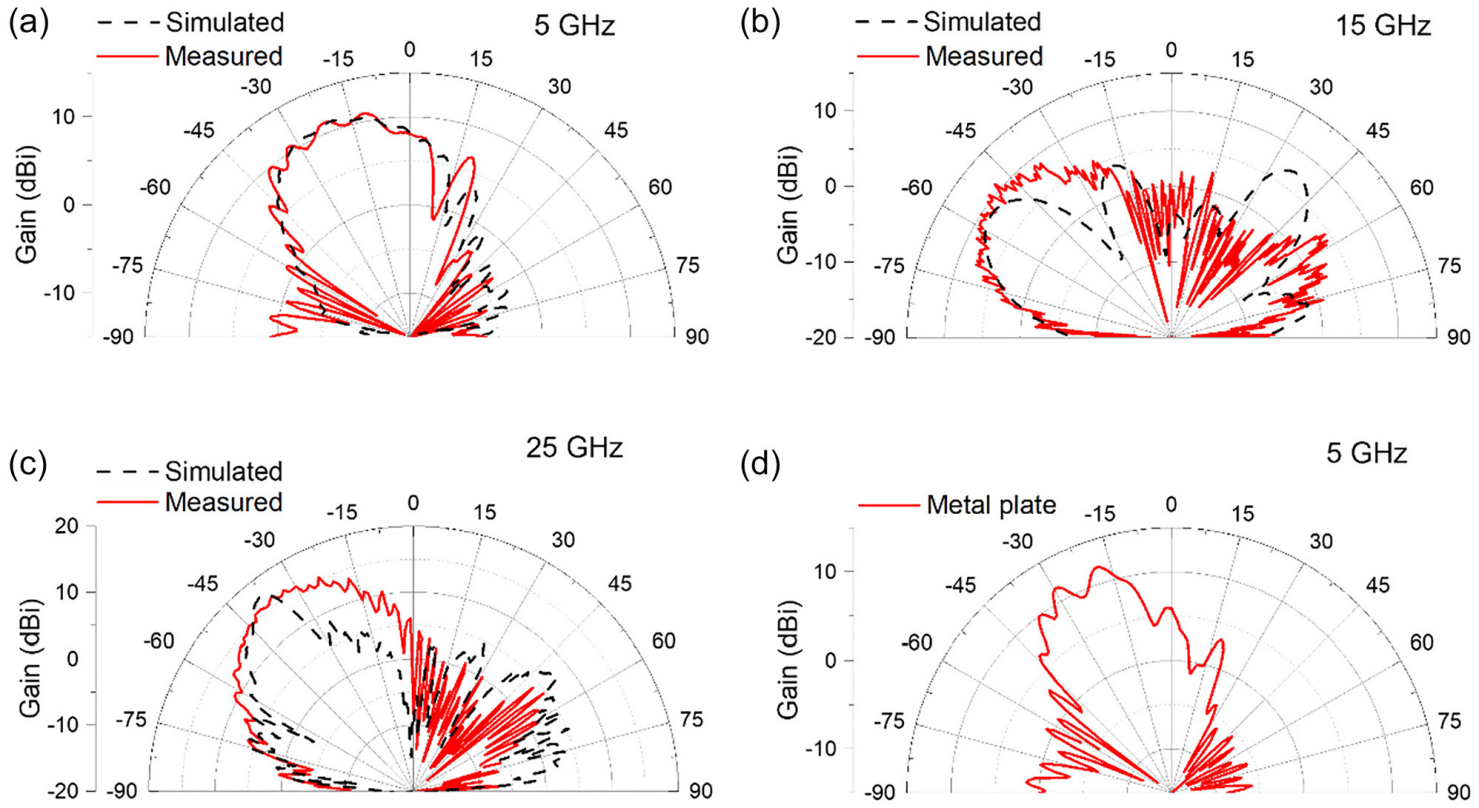
Experimental characterization of the multi-frequency beam steering metasurface with an incidence angle of 20°. (a)–(c) Simulation (black) and experimental (red) results of far-field radiation patterns at (a) 5 GHz, (b) 15 GHz, and (c) 25 GHz, respectively, under *x*-polarized wave excitation. (d) Experimental result of far-field radiation pattern of a metal plate with an incidence angle of 20°.

The simulated and experimental results of multi-frequency beam steering metasurface at 5 GHz, 15 GHz, and 25 GHz are plotted in Figure 5. In the measurement, the polarization state of receiver horn antenna (Rx antenna) was fixed to *x*-polarized for 5 GHz measurement and fixed to *y*-polarized for 15 GHz and 25 GHz measurement. The red curve and black dotted curve represent measured and simulated far field radiation pattern from the metasurface, respectively. The incident angle of Gaussian beam from Tx antenna was set to 20°, therefore the calculated beam steering angles of the reflected beams from the metasurface were −20°, −50° and −37° for 5 GHz, 15 GHz, and 25 GHz, respectively. In numerical simulation, input sources exported from the horn antenna used in the measurements were used for correct and direct comparison between the simulated and measured results. At 5 GHz, the peak intensity of the reflected beam with *r*_*xx*_ component was measured at an angle of about *θ* = −20°, which is equal to the angle of incidence as shown in Figure 5(a). We conducted the same measurement using a simple metal plate as shown in Figure 5(d), exhibiting the similar far field intensity pattern. It confirmed that the metasurface at 5 GHz is working as a metallic mirror. At 15 GHz and 25 GHz, the peak intensity of the reflected beams with *r*_*xy*_ component was measured at angles of about *θ* = −50° and −36°, respectively, as shown in Figure 5(b) and (c). All the measured results were matched very well with the results of simulation. Compared to the calculation results in Figure 3, the secondary lobes were measured to be large, which is the result of including the near-field component because the distance between the Tx and Rx antennas was not sufficiently separated. From the measurement results, different reflected wave beam steering angles were formed at fundamental frequency 5 GHz and THG and FHG frequencies 15 and 25 GHz. It was confirmed that it operates as a multi-frequency spatial filter through the characteristic. The metasurface proposed in this study can be easily extended to higher frequency domains, and to demonstrate this, numerical simulations were performed in the 0.16 to 0.8 THz region. The simulation results are provided in Supplementary Material Figure S1 and S2.

## 4 Conclusions

In this work, we have proposed and experimentally demonstrated multi-frequency beam steering metasurface for producing multi-frequency spatial filtering using the optimized split ring structure arrays. The polarization convert meta-atoms have the capacity of engineering local phase and amplitude profiles with extremely broadband that can cover fundamental frequency to fifth harmonic frequency. By arraying the designed meta-atoms, FF signals and high odd-harmonic signals are de-multiplexing to different direction at each frequency. The proposed metasurface can operate in a frequency range of more than 2 octaves, and this feature has never been implemented in previous multi-frequency metasurface studies (cf. Supplementary Material Table S2). The proposed approach in this work will provide an efficient device platform for potential applications, such as multi-frequency beam shaping, spatial filter for harmonic generations, and multi-frequency target tracking. In addition, the proposed approach may be applied as a spatial filter for multiple harmonic generation signals in the sub-terahertz or terahertz domain, which is higher frequency region, through structure optimization and scaling down.

## Supplementary Material

Supplementary Material Details
