# Supplementary material for: Metasurface spatial filters for multiple harmonic signals
Source: Nanophotonics. 2023 Mar 31;12(13):2397–403. doi: 10.1515/nanoph-2022-0752 (PMC11501340; doi:10.1515/nanoph-2022-0752)
Supplement: Supplementary file 1 — Supplementary Material Details [file j_nanoph-2022-0752_suppl_001.docx]

Supplementary Material for

**Metasurface spatial filters for multiple harmonic signals**

Daeik Kim, Mau Anh Nguyen, Gangil Byun and Jongwon Lee^*^

Department of Electrical Engineering, Ulsan National Institute of Science and Technology (UNIST), Ulsan 44919, Republic of Korea

* Corresponding Author: Jongwon Lee (E-mail: [jongwonlee@unist.ac.kr](mailto:jongwonlee@unist.ac.kr))

**Properties of the co- and cross-polarized reflection coefficients.**

As shown in Table 1, four of two pairs of meta-atoms (1&5, 2&6, 3&7, and 4&8) have the same shape with a different in-plane orientation angle of 2α = 90°. More specifically, Jones matrix for the rotation of each pair can be described as:

$R(\theta=2\alpha=90^{\circ})=\left[\begin{matrix} \cos\theta& -\sin\theta\\ \sin\theta& \cos\theta\end{matrix}\right]\left[\begin{matrix} r_{xx} & r_{xy} \\ r_{yx} & r_{yy} \end{matrix}\right]\left[\begin{matrix} \cos\theta& \sin\theta\\ -\sin\theta& \cos\theta\end{matrix}\right]=\left[\begin{matrix} r_{yy} & -r_{yx} \\ -r_{xy} & r_{xx} \end{matrix}\right]$ (S1)

where $\theta$ is the rotated angle. From symmetry of our meta-atom structure, co- and cross-polarized reflection coefficients of each meta-atom have the characteristics of $r_{xx}=r_{yy}$and $r_{xy}=r_{yx}$. Therefore, for each pair of two meta-atoms, the co-polarized reflection coefficient is $r_{xx}$, which has the same magnitude and phase value, and the cross-polarized reflection coefficient has the same magnitude as $r_{yx}$, but there is a phase difference by π.

**Design and simulation for the metasurface spatial filters for multiple harmonic signals at THz region.**

To confirm that the proposed approach can be applied as a spatial filter for multiple harmonic generation signals in the sub-terahertz or terahertz (THz) domain, eight meta-atoms were designed, and numerical simulation was conducted at THz region. The split ring resonator arrays were patterned on a dielectric substate layer on a metal back plane in the same way of the metasurface in manuscript. The length of unit cell is 150 μm and thickness of metal is 18 μm The dielectric substrate material was Taconic TLT-9 with a dielectric constant of 2.5, a loss tangent of 0.0009, and thickness of 50 μm. The Table S1 provide the detail design parameter of the eight meta-atoms.

**
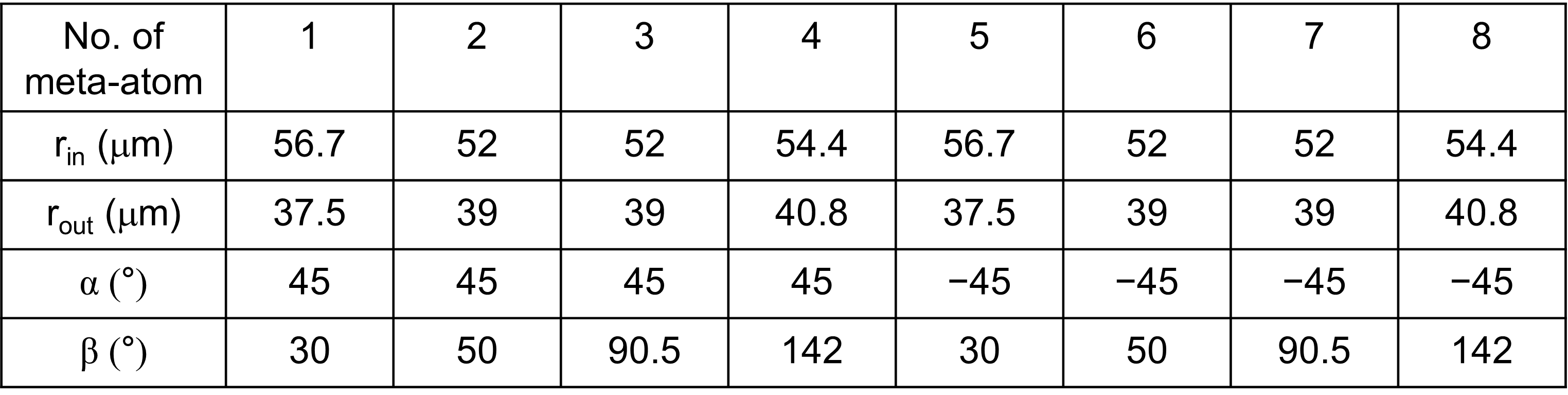
**

**Table S1.** Design parameters of the eight meta-atom unit structures for THz region.

Figure S1 shows the simulated local phase and reflection coefficient of the designed meta-atoms using by Commercial Software package (CST Microwave Studio) at THz region. Figure S1a and S1b show simulated magnitude and phase of reflection coefficients of the eight meta-atoms under the x-polarized input and the same polarized reflection output, indicated as r_xx_. In case of the r_xx_ at 0.16 THz, the reflection coefficients are nearly unity and reflection phase values are the same for the eight meta-atoms. Therefore, the super cell acts as a simple metallic mirror, leading to specular reflection at 0.16 THz for the r_xx_ component. Figure S1c and S1d show simulated magnitude and phase of reflection coefficients of the eight meta-atoms under the x-polarized input and y-polarized reflection output, indicated as r_yx_. In case of the r_yx_, reflection coefficients are over 0.86 and 0.8 at 0.48 THz and 0.8 THz, respectively, for the eight meta-atoms and local phase values form eight different values with nearly 45 degrees of spacing at the two harmonic frequencies. Therefore, any arbitrary wavefront engineering is possible by using the eight-phase element meta-atoms at the two harmonic frequencies. According to the Equation (1), the calculated beam steering angles for the two harmonic frequencies using the equation (1) were $31^{\circ}$ for 0.48 THz and $18^{\circ}$ for 0.8 THz.

To confirm the theoretical calculation results, far-field radiation pattern simulations were conducted under a normal incidence of a plain wave. Figure S2 shows two-dimensional cross-section of simulated far-field radiation patterns at 0.16 THz, 0.48 THz, and 0.8 THz. For clear identification, the intensity radiation patterns at each frequency were linearly normalized to the respective maximum intensity value. For normal incident waves at 0.16 THz, 0.48 THz, and 0.8 THz the beam angles reflected from the metasurface are steered to 0$^{\circ},$ 30$.4^{\circ}$ and 17.9$^{\circ}$, respectively. The numerical simulated and calculated results are matched very well.

**
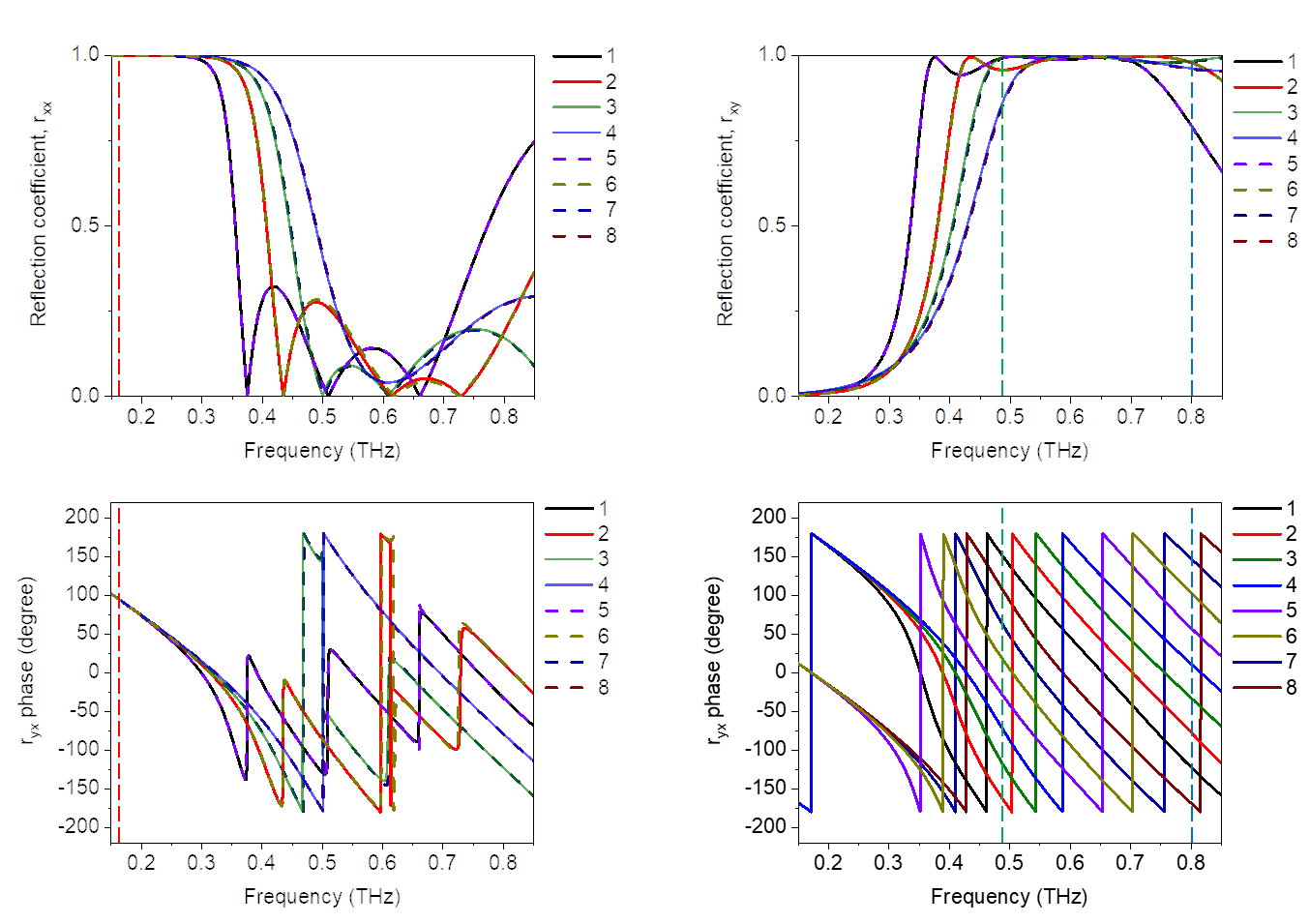
**

**Figure S1.** Simulated reflection coefficients of the eight meta-atoms at THz region. (a, b) Simulated (a) magnitude and (b) phase spectra of reflection coefficient for x-polarized reflection beam under the x-polarized incident beam, indicated as r_xx_. (c, d) Simulated (c) magnitude and (d) phase spectra of reflection coefficient for y-polarized reflection beam under the x-polarized incident beam, indicated as r_yx_.


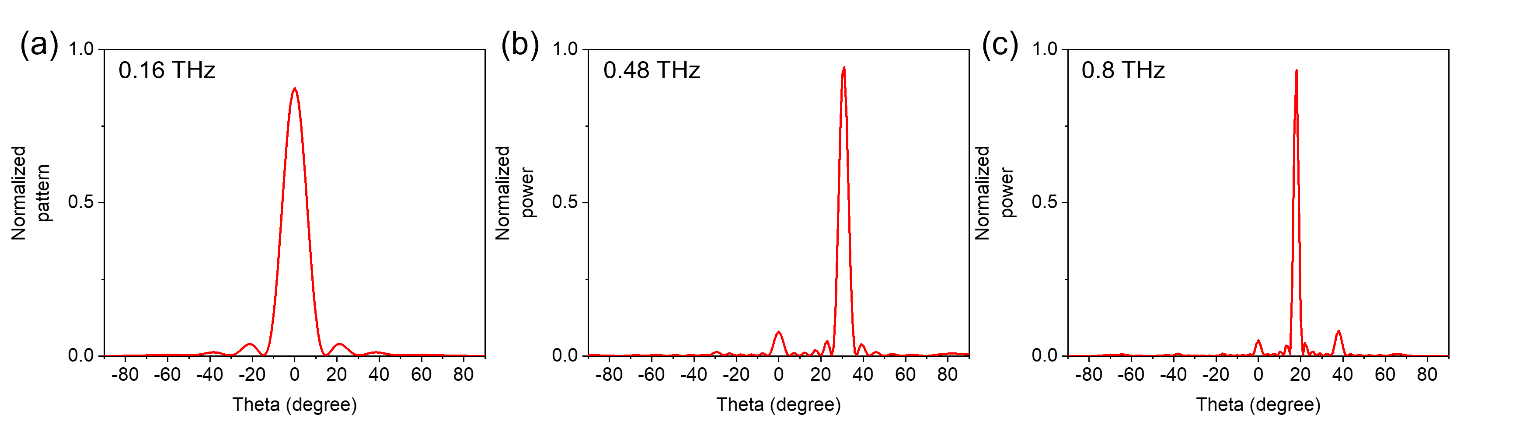


**Figure S2.** Simulation results of far-field scattering patterns from the metasurface. (a-c) two-dimensional cross-section of the scattered intensity patterns at (a) 0.16 THz, (b) 0.48 THz and (c) 0.8 THz under normal incidence wave.

**
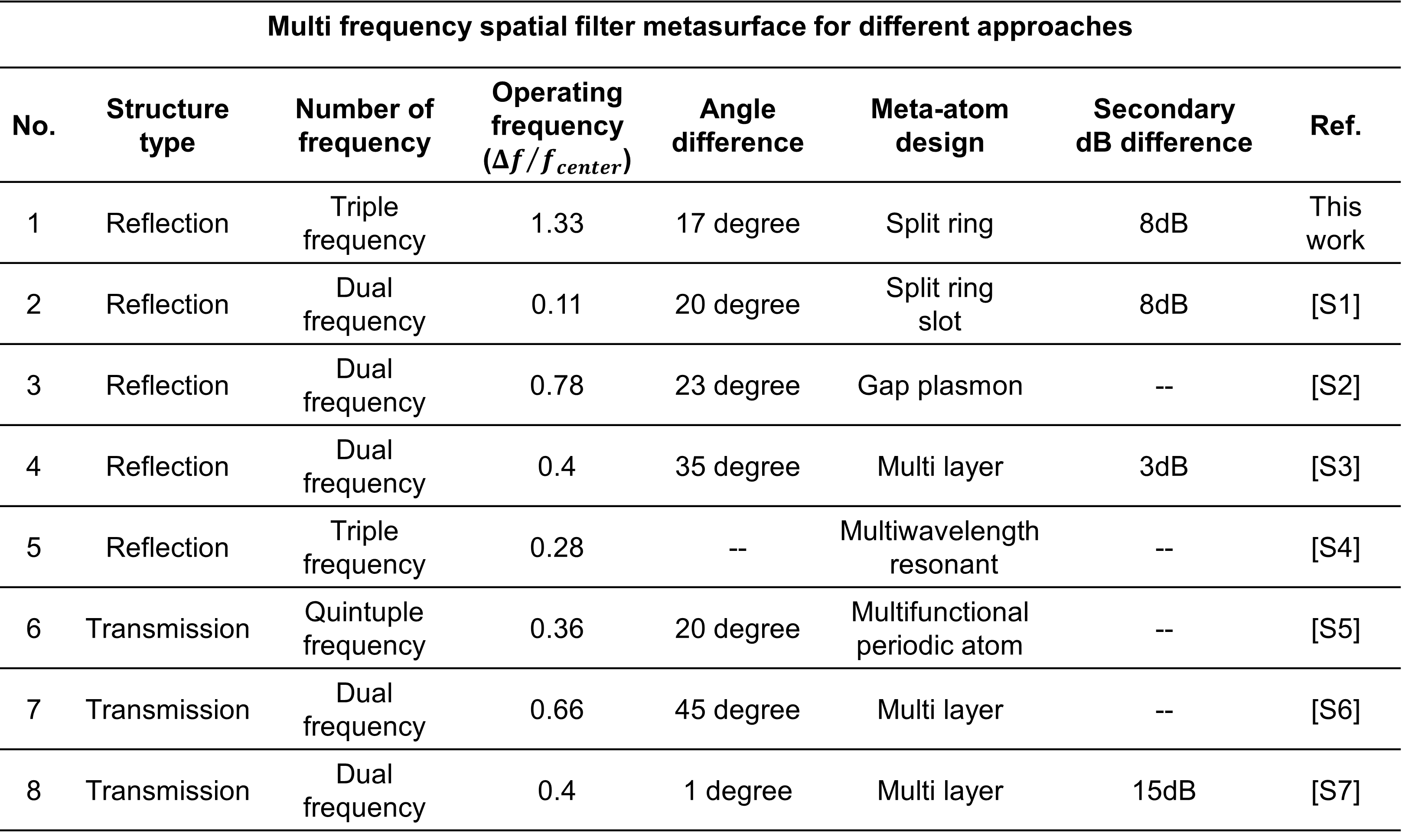
**

**Table S2.** Comparative table of multi frequency beam steering metasurface performance for different approaches. The operating frequency is defined as $\frac{\Delta f}{f_{center}}$, where $\Delta f$ is a bandwidth ($f_{max}-f_{min}$) and $f_{center}$ is a center frequency of the metasurface. The secondary dB difference is defined as minimum value of $G_{first}-G_{secondary}$, where $G_{first}$ is gain of first lobe and $G_{secondary}$ is gain of secondary lobe.

**Supplementary references**

[S1] Jesus A., *et. al*, “Dual-Frequency Reflect array Based on Split-Ring Slots”-IEEE Antennas and Wireless propagation, **16** (2016)

[S2] Rucha A., *et. al*, “Dual-Band Metasurfaces Using Multiple Gap-Surface Plasmon Resonances”, Appl Mat & Int, **12** (2020)

[S3] Shahid I., *et. al*, “Dual-band 2-bit coding metasurface for multifunctional control of both spatial waves and surface waves”, Journal of OSA B, **36** (2019)

[S4] Adam C., *et. al*, “Multifunctional Nonlocal Metasurfaces”, PRL, **125** (2020)

[S5] David S., *et. al*, “Periodic Dielectric Metasurfaces with High-Efficiency, Multiwavelength Functionalities”, AOM, 16 (2017)

[S6] Zhenkun L., *et. al*, “Anomalous wavefront control via nonlinear acoustic metasurface through secondharmonic tailoring and demultiplexing”, Appl Phys Lett, **121** (2022)

[S7] H. Hasani., *et. al*, “Dual-Band Circularly Polarized Transmitarray Antenna for Satellite Communications at (20 to 30) GHz”, IEEE TRANSACTIONS ON ANTENNAS AND PROPAGATION, **67** (2019)
